# Supplementary figures and images for: Attenuation hotspots in neurotropic human astroviruses
Source: PLoS Biol. 2023 Jul 17;21(7):e3001815. doi: 10.1371/journal.pbio.3001815 (PMC10374088; doi:10.1371/journal.pbio.3001815)

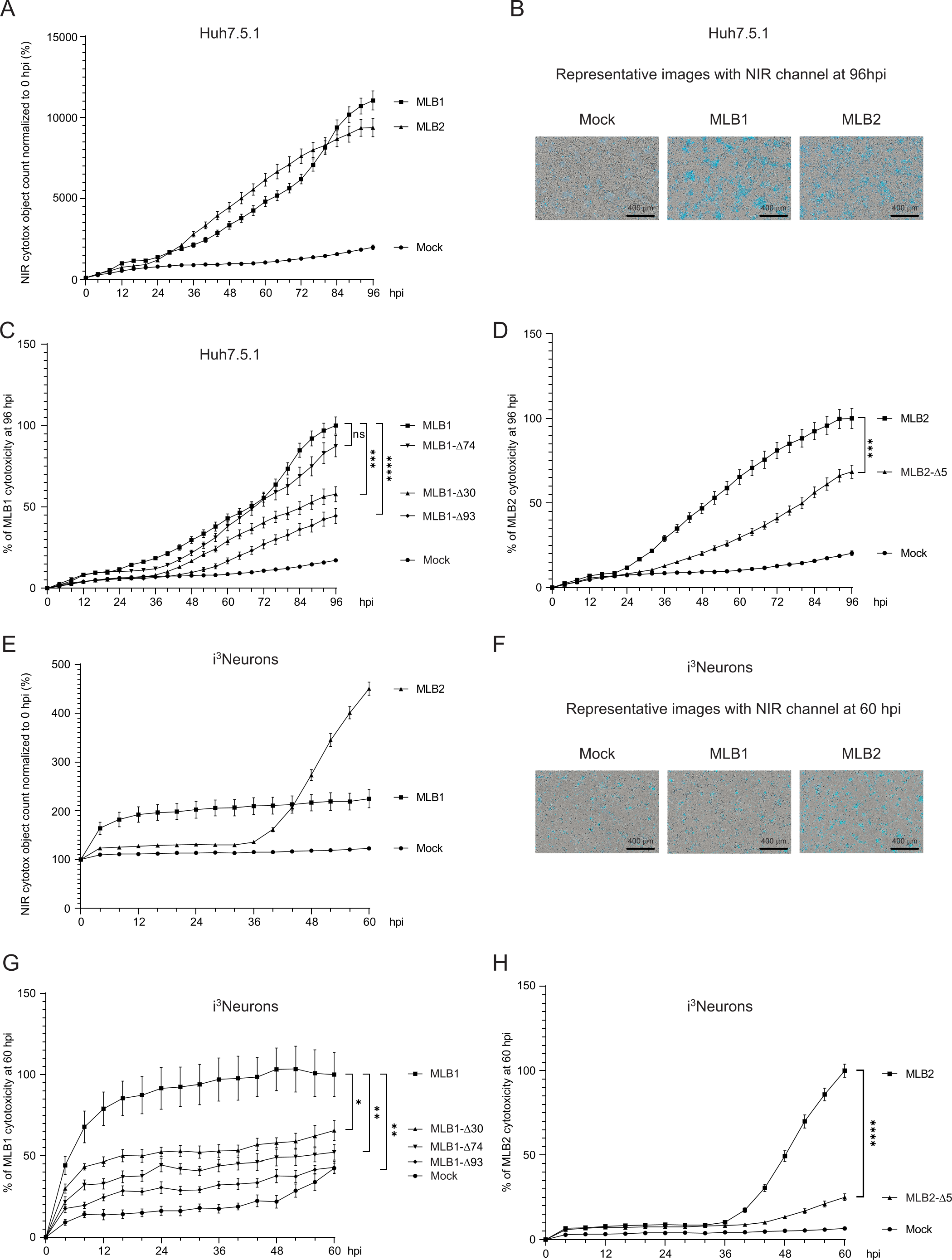

Supplement: S1 Fig — Terminally differentiated neurons and Huh7.5.1 cells grown on 96-well plates were infected at MOI 0.5 in quintuplicates. Cytotoxicity was assessed by live-cell imaging using Cytotox NIR Dye added directly during virus infection. (A) NIR object counts data for Huh7.5.1 cells infected with MLB1 and MLB2, normalized to 0 hpi. (B) Representative images of MLB-infected Huh7.5.1 cells at 96 hpi. (C) Cytotoxicity analysis of MLB1 and mutant infected Huh7.5.1 cells, normalized to maximum MLB1 toxicity. (D) Cytotoxicity analysis of MLB2 and mutant infected Huh7.5.1 cells, normalized to maximum MLB2 toxicity. (E) NIR object counts data for i3Neurons infected with MLB1 and MLB2, normalized to 0 hpi. (F) Representative images of MLB-infected i3Neurons at 60 hpi. (G) Cytotoxicity analysis of MLB1 and mutant infected i3Neurons, normalized to maximum MLB1 toxicity. (H) Cytotoxicity analysis of MLB2 and mutant infected i3Neurons, normalized to maximum MLB2 toxicity. All data are mean ± SEM (n = 5), ns, nonsignificant, *p < 0.05, **p < 0.01, ***p < 0.001, ****p < 0.0001 using two-way ANOVA with repeated measures test against wt MLB infection. All individual quantitative observations that underlie the data can be found in S1 Data file. (TIF) [file pbio.3001815.s001.tif]
