# Supplementary material for: Attenuation hotspots in neurotropic human astroviruses
Source: PLoS Biol. 2023 Jul 17;21(7):e3001815. doi: 10.1371/journal.pbio.3001815 (PMC10374088; doi:10.1371/journal.pbio.3001815)

Figure 2B

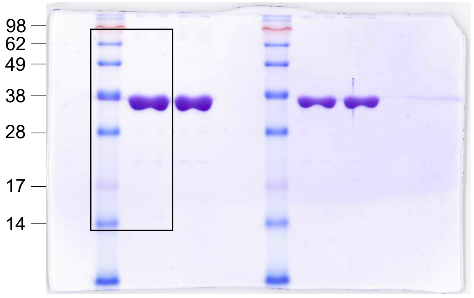

Figure 2C

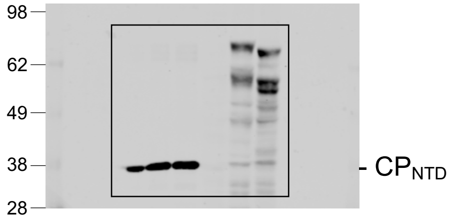

anti-CP

Figure 2C

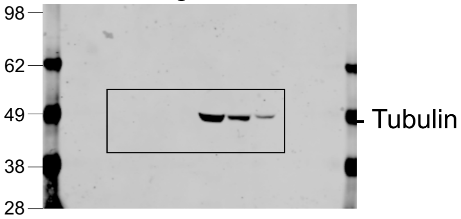

anti-tubulin

Figure 3F

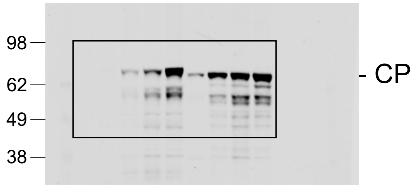

anti-CP

Figure 3F

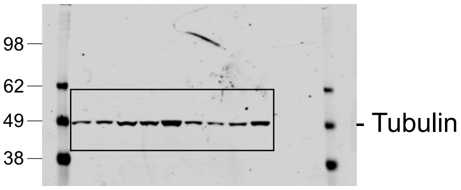

anti-tubulin

Figure 3G

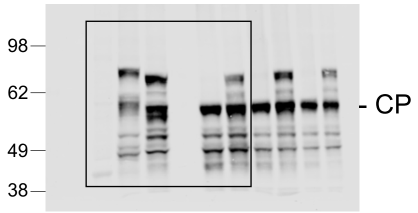

anti-CP

Figure 3G

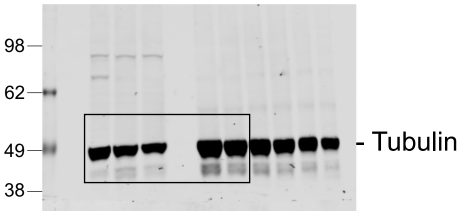

anti-tubulin

Figure 3J

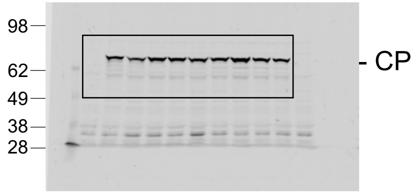

anti-CP

Figure 3J

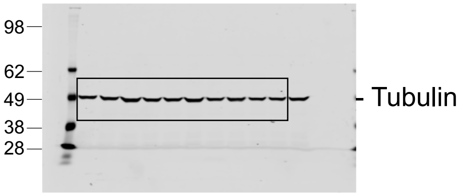

anti-tubulin

Figure 3K

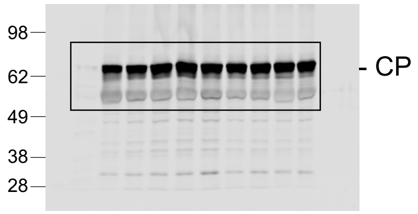

anti-CP

Figure 3K

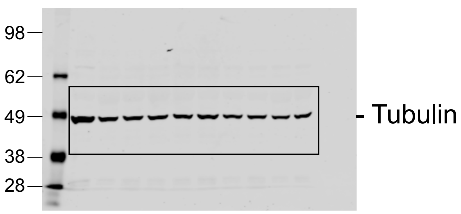

anti-tubulin

Figure 4E

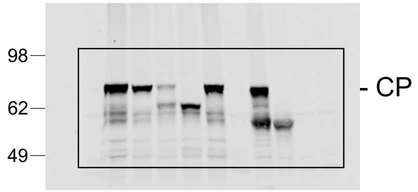

anti-CP

Figure 4E

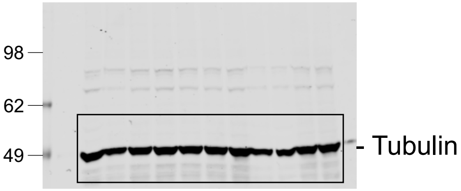

anti-tubulin

Figure 4F

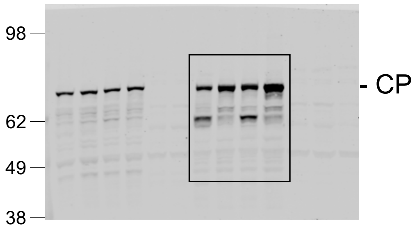

8E7 anti-HAstV CP

Figure 4F

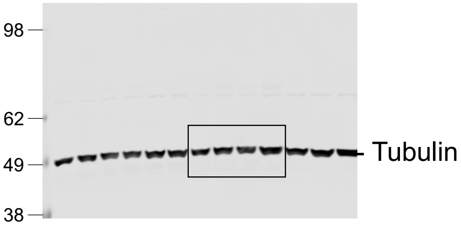

anti-tubulin

Figure 4F

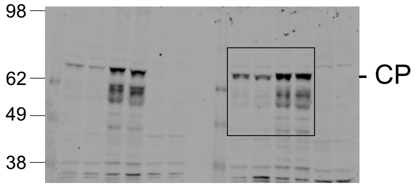

anti-CP

Figure 4F

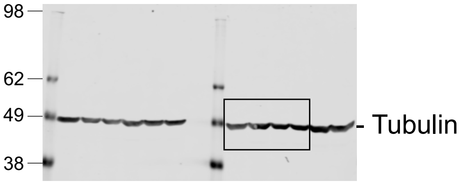

anti-tubulin

Figure 4F

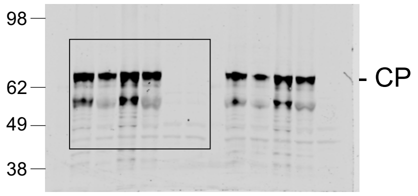

anti-CP

Figure 4F

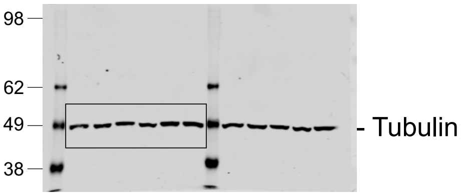

anti-tubulin

Figure 4H

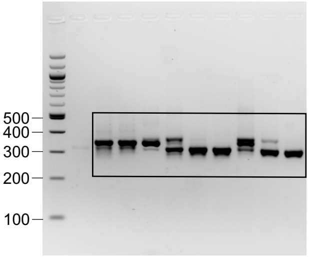

Figure 4H

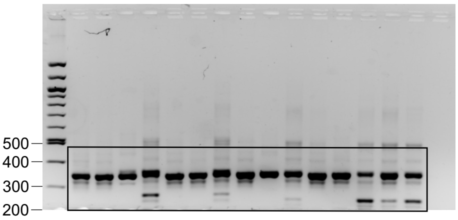

Figure 4H

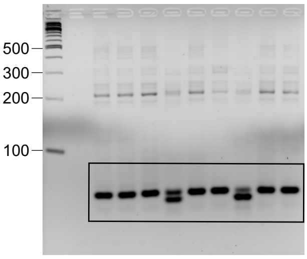

Supplement: S1 Raw Images — (PDF) [file pbio.3001815.s003.pdf]
