# Supplementary material for: Attenuation hotspots in neurotropic human astroviruses
Source: PLoS Biol. 2023 Jul 17;21(7):e3001815. doi: 10.1371/journal.pbio.3001815 (PMC10374088; doi:10.1371/journal.pbio.3001815)
Supplement: Attachment — Submitted filename: Responses to reviewers 2023-04-16.docx [file pbio.3001815.s004.docx]

**Manuscript PBIOLOGY-D-22-01870R1**

**Note from the editor**

Having discussed the reviews with the Academic Editor, we think that it is important for you to demonstrate the neural tropism of the viruses and the utility and faithfulness of the recombinant system. It would be particularly compelling if the results are validated by real world phylogenetic comparisons, if the sequences exist. If not, passaging in neuronal cells and fortifying those experiments would be needed.

We thank the editorial team for this suggestion. In the revised manuscript, we have demonstrated neural tropism by serially passaging viruses and mutants in iPSC-derived neurons, confirming the ability of recombinant WT but not attenuated mutant viruses to productively replicate in mature neurons. Unfortunately, sequences that would be useful for the suggested phylogenetic analysis do not exist. The deletions we observed were specific to attenuated viruses and were not observed in natural isolates. We further confirmed this by sequencing WT viruses that were passaged in neurons.

**Response to Reviewers**

We thank all reviewers for their thorough and thoughtful comments on the manuscript. We have carefully considered all the comments and have now provided the requested experimental and analytical analyses, in addition to further clarification, where appropriate.

**Reviewer #1**

In this manuscript, Ali et al undertake an ambitious and comprehensive series of experiments to define regions of the astrovirus MLB genome required for replication in neuronal cells. The authors take a directed evolution approach to adapt clinical MLB isolates and then develop a battery of tools to identify the genomic changes associated with replication in cells including human i3 neurons. Yet, the manuscript primarily focuses on tool development/ characterization and using their newly described reverse genetics system to assess genomic stability, analyze naturally occurring mutations in the genomes, and creating recombinant viruses with deletions to assess growth in Huh7.5 cells. There are minimal studies characterizing growth in neuronal cells and no evidence that MLB1 or 2 productively replicates in these cells. This is a major concern about the studies. Further, replication in a single neuronal cell ex vivo fails to address neurotropism. Based on the presented data, the authors are strongly encouraged to refocus the manuscript on characterization of the new reverse genetics system and understanding the areas of the genome involved in replication in Huh7.5 cells, which is the strongest part of the studies.

We thank the reviewer for their comments. We discussed the methodology-heavy aspect of our manuscript with the editor and they suggested considering it for the “Methods and Resources” section, thus justifying this aspect. In addition, the characterization of viral growth in neuronal cells has been substantially improved in the revised version of the manuscript. We have demonstrated that WT MLB viruses, but not attenuated mutant viruses, can productively replicate in neuronal cells. Detailed explanations and specific comments are provided below.

Major

1. The majority of the studies focus on understanding adaptations generated in serially passaging MLB1 and MLB2 co-infected Huh7.5 cells. There is no discussion on why co-infections are used. There is no evidence from human studies that MLB1 and 2 co-infect people. It is also unclear why both strains are used rather than focusing on the more frequently isolated MLB1 strain.

Co-infection is a well-known evolutionary strategy based on complementation of replication-compatible genomes. It is not based on potential natural co-infections, but is purely an in vitro approach for creating favorable conditions for less “fit” genomes. Co-infection provides an opportunity for viral genomes to interact through complementation and stimulate the evolution process. We have expanded this section and provided the corresponding references (lines 105-125 in the revised manuscript).

The use of both the MLB1 and MLB2 genotypes allowed us to generalize our conclusions. The independent occurrences of similar evolutionary adaptations in two different genotypes in several different experimental setups allow us to draw a confident conclusion regarding attenuation strategies in neurotropic MLB astroviruses.

2. Is there evidence from comparative phylogenetic studies, that the genomes of CNS-isolated MLB strains differ from those obtained from the intestines? Given that these viruses are a major cause of CNS-associated infections in several animal species, these sequences should be available for analysis.

We were unable to identify studies in which such comparative analyses were performed, it would be indeed interesting if they were available. Most genomic analyses have been based on CNS-associated outbreaks. We performed a comparative analysis of all available MLB strains (Fig. 4A-B) and did not find any deletions in the attenuation region, suggesting it is dispensable only in laboratory settings, e.g. during passaging in susceptible cell lines. Other differences may indeed be possible, but the current stage of astrovirus research cannot address their significance. A more detailed understanding of the genome organization and functions of individual elements (proteins and RNA elements) is required to understand and predict the impact of individual genetic changes.

3. More information is needed on the source of the clinical isolates used and whether they were derived from enteric or CNS infection.

We thank the reviewer for this suggestion. We have expanded this section, as suggested (lines 110-112 in the revised manuscript). The exact details can be found in a previously published study (Vu *et al*., JVI, 2019, PMID 31019055).

4. A major premise of the study is identifying the regions of the viral genome associated with neurotropism. The author's tackle this by adapting clinical MLB viruses in cell culture and constructing recombinant viruses to study replication in particular neuronal cells in culture. Yet, the adaptation takes place in non-neuronal, non-biologically relevant Huh 7.5 cells. It is unclear how this would translate the in vivo situation. These studies should be repeated in neuronal cells to assess the molecular changes that occur.

We thank the reviewer for the suggestion. To demonstrate the stability of MLB astroviruses in neuronal cells, we have passaged both WT and mutant viruses in neurons and significantly expanded this section of the manuscript (Fig 6 and Fig S1).

We also expanded the explanation of why passaging in Huh7.5 cells was used. Adaptation of viruses in susceptible cell lines or other immune-deficient models (such as embryonated eggs or neonatal mice) is a widely used strategy to create attenuated viruses and vaccine candidates (PMID 25136134). These cells or model organisms do not have to be biologically relevant as long as they support virus replication and lead to virus attenuation due to the lack of a full immune response (lines 105-110 in the revised manuscript). We tested these viruses in the context of biologically relevant iPSC-derived neurons, confirming that the deletions observed in Huh7.5 cells are not beneficial for the viruses in this system (lines 424-430 and expanded Fig 6 in the revised manuscript).

5. The authors fail to demonstrate that MLB viruses productively replicate in neuronal cells in vitro. Further, the references cited do not support their claim that these viruses infect cells of neuronal origin.

We thank the reviewer for the valuable comment. The characterization of viral growth in neuronal cells has been substantially improved in the revised version of the manuscript. We have demonstrated that WT MLB viruses, but not attenuated mutant viruses, can productively replicate in neuronal cells. We performed several successful reinfection experiments using viruses isolated from neuronal infections. Furthermore, we titrated these neuron-passaged viruses and confirmed their efficient spread and ability to infect naïve neurons.

To demonstrate the neurotropic potential of MLB astroviruses, we have cited the following studies: PMID 27088842, 26991054 (Introduction), 28820378, 24989603, and 20926705 (Discussion). Evidence of specific infection of neuronal cells is provided in the current manuscript and is supported by co-staining of MLB-specific antibody with the mature neuron-specific marker MAP2 (Fig 6).

Reviewer #2: This article describes a novel reverse genetics system that should help us understand the biology of the MLB genogroup of astroviruses. The main advantage over previous systems is that transfection, recovery, and subsequent viral propagation are performed in the same cell line, as opposed to previous systems using two cell lines (one for recovery and another for propagation). The article is essentially methodological and, although the proposed techniques should be relevant, no major advances in our understanding of these astroviruses is reported here. I thus find it questionable whether this manuscript brings sufficient advance for publication in a generalist journal such as PLoS Biol, or should be preferably published in a more specialized journal in the field of virology.

We thank the reviewer for the constructive criticism. We respectfully disagree that our study did not provide sufficient advances in astrovirus biology. To the best of our knowledge, this is the first report demonstrating the ability of MLB astroviruses to infect mature neurons and the first report demonstrating regions in the genome responsible for attenuation. Furthermore, we have provided a detailed analysis of recombinant attenuated viruses in the Huh7.5.1 cell line and during neuronal infection. This expands our understanding of the neurotropic determinants of pathogenic RNA viruses.

Specific comments:

1- To better appreciate the advantages of the reverse genetics system developed here, the authors should further discuss the limitations of the previous strategies. Given that recovery is apparently straightforward (transfection of a T7 in vitro transcript), why the previous systems cannot be implemented in a single cell line?

We thank the reviewer for this suggestion and have amended the revised manuscript accordingly (lines 200-203).

2- How was the sequencing performed? Specifically, how were the 5´and 3´ genome ends sequenced?

We thank the reviewer for pointing out the missing details and have now specified the details in the Materials and Methods section: “Virus RNA was isolated using Direct-zol RNA MicroPrep (Zymo Research), followed by RT-PCR and Sanger sequencing of the virus genome. The genome ends were sequenced using 5'/3' RACE kit (Roche)” (lines 612-614 in the revised manuscript).

3- lines 113-120: I don´t see how these two viruses could complement if the MOI was 0.1.

We understand the reviewer’s reservations regarding the MOI used in this study. The rationale for starting infection with a lower MOI was dictated by longer cell survival during the initial infection (see Fig 2E for reference when MOI 1 was used). This allows productive infection with several replication cycles, which eventually results in co-infection and complementation.

4- Why was the recovered virus stable in permissive cells (Huh7.5), whereas the clinical isolates evolved more mutations? Information about how these clinical isolates were passaged is missing.

We thank the reviewer for pointing this out. This was observed only for MLB2 virus. Reverse genetics-derived MLB2 was very stable and did not result in any mutations after 10 passages in three parallel experiments. The appearance of a single deletion (MLB2-Δ5) upon passaging of the initial clinical isolate may be due to the quasispecies-nature of clinically isolated viruses. This has now been discussed in the revised manuscript (lines 463-464).

5- The deletions that emerged in the serially passaged viruses should be beneficial under the conditions of this evolution experiment, unless there is a specific reason for their emergence other than positive selection. Could the authors perform competition assays to test this? This benefit is suggested by the replicon experiments, but could be further shown using full viruses. Interestingly, the mutants seem to exhibit accelerated replication in neurons but an overall fitness cost due to deficient virus gene expression, assembly or release. It would be interesting to also have data on replication kinetics and total viral fitness in the permissive cell line to more precisely identify the stage of the infection cycle where differences between Huh7.5 cells and neurons take place.

We thank the reviewer for the excellent suggestions. We have performed both experiments and the results have significantly improved our understanding of mutant virus behavior and their selective advantage over their WT counterparts in different tested systems (replicon versus virus infection in Huh7.5.1, and virus infection in neurons).

The virus competition experiment demonstrated a clear fitness advantage for MLB1-Δ30 and the ability to co-exist with wt for MLB1-Δ93 (both are in-frame deletions), but not for MLB1-Δ74 and MLB2-Δ5 (both are out-of-frame deletions) – see updated Figure 4H and lines 314-328 in the revised manuscript. This suggests that the C-terminal part of the capsid polyprotein is important in the context of the full virus infection process (assembly/packaging) but dispensable in the replicon system. This significantly improved our understanding of the RNA/protein phenotypic differences in deletion mutants.

We also performed virus replication kinetics experiments and RNA analyses in Huh7.5.1 cells. The changes are introduced as follows:

- Fig 4D, S1 and lines 282-291 in the revised manuscript (growth kinetics of WT and mutants).
- Fig 5F-G and lines 385-391 in the revised manuscript (RNA levels in infected Huh7.5.1 cells).

Reviewer #3: The manuscript by Ali et al. provides an exciting new advance to the study of human astroviruses, MLB1 and MLB2, in the form of molecular clones and replicon systems. With these new systems the authors define a difference in genomic stability between the two viruses as well as a difference in reliance on caspase cleavage for capsid production. Furthermore, this study demonstrates the novelty of using replication deficient astroviruses as a means to drive attenuation and the prospect of developing live-attenuated strains for vaccines. Overall the data and experimentation are clear and provide sound conclusions. However, the following suggestions would help clarify a few key points in the manuscript:

We thank the reviewer for the optimistic summary and are happy to address the specific questions.

Major

Revisions of Fig1a and accompanying figure legend should be considered since most, or possibly all, sequences in the tree were obtained from stool samples collected during gastrointestinal illness. Therefore, it is difficult to assign strictly neuronal tropism to all members of those clades. Additionally, 2 classical HAstV cases of neurological disease have been identified (Koukou 2019, Wunderli 2011) but the neuron picture was not included for that clade.

We thank the reviewer for their comment. We have modified Fig 1A and the legend, specifying that some of the astrovirus genotypes labeled with the neuron icon were associated with neurotropic manifestations. It remains to be seen what proportion of these viruses is associated with neurotropic diseases once more clinical/epidemiological data become available. We have added the neuron icon to HAstV1 and HAstV4 following the reviewer’s advice and mentioned these cases in the related section of the discussion (lines 482-483).

In the competition experiment (Fig 1c), it is noted that there was no evidence of out-competition for 10 consecutive passages. As a point of clarification- were the same levels (ie. titer) of the virus found after each passage or were the titers after each passage not significantly different than when the viruses are passaged separately?

We thank the reviewer for this comment. Owing to the cross-reactivity of capsid-specific antibodies, we were not able to distinguish the actual titers in the mixed samples. The analysis of viral RNAs by qPCR is not indicative of actual titers due to abnormal RNA replication levels in viruses with deletions (Fig 4D and 5F-G). We did not notice any changes in the amount of MLB-specific RT-PCR products, showing, although non-quantitatively, that both viruses were present in the competition/complementation experiment during passaging.

Fig 6G and the accompanying data convincingly show that the deletion mutants result in much higher ratios of defective particles. Was there evidence of this during the sequencing of passaged viruses?

We thank the reviewer for this note. To make the results from neurons and Huh7.5.1 infections comparable, we replaced previous RNA analyses with direct comparative RNA levels (Fig 5F-G and 6G). These data show the unbalanced RNA synthesis in both systems, leading to increased levels of defective particles that are not viable in neuronal infections on passaging (Fig 6E). Evolution experiments have resulted in a mixed phenotype (due to multiple genome sequences present) that was hard to link to a particular sequence/virus.

Line 422: While trypsin is typically thought as being gut-specific, the brain also houses several trypsin-like proteases. Therefore, this explanation for extra-gastrointestinal disease associated with MLB and VA1 strains may be insufficient.

We thank the reviewer for this note. We have modified our statement to accommodate other scenarios (lines 479-483).

While the attenuation noted in Fig6 indicates reduced infection, the presence of virus particles, including defective particles, may still trigger inflammation and cell death of neurons. Such experiments may be needed in future evaluation of live-attenuated strains for vaccine development.

We thank the reviewer for the valuable suggestion. We have performed cell viability experiments in both neurons and Huh7.5.1 cells for all mutants and WT viruses (Fig S1). All mutant viruses were less cytotoxic despite a higher proportion of defective particles. This further confirmed their potential for vaccine development.

Minor

Can the authors include the passage number that was used to obtain the data in Fig3D and E?

Yes, thank you for the suggestion (line 224 in the revised manuscript).

Line 160: was it previously shown that passaging the virus in Huh7.5.1 results in enhanced replication and CPE compared to Huh7?

No, this has not been observed before. A previous study reported that Huh7 and Huh7.5 cells support persistent infection (PMID: 31019055). In all our experiments, mutant viruses consistently resulted in reduced CPE compared to WT (Fig S1).

Line 240: Point of clarification- "…all related WT MLB astrovirus sequences" were those available in GenBank?

Thank you for this suggestion. We have modified the sentence for clarity (line 249 in the revised manuscript).
