# Supplementary material for: Attenuation hotspots in neurotropic human astroviruses
Source: PLoS Biol. 2023 Jul 17;21(7):e3001815. doi: 10.1371/journal.pbio.3001815 (PMC10374088; doi:10.1371/journal.pbio.3001815)
Supplement: Attachment — Submitted filename: Responses to reviewers and editor 2023-05-26.docx [file pbio.3001815.s005.docx]

**Manuscript PBIOLOGY-D-22-01870R2**

**Response to the editor**

Based on the reviews and on our Academic Editor's assessment of your revision, we are likely to accept this manuscript for publication, provided you satisfactorily address the remaining points raised by reviewer #2. To address the remaining concerns from reviewer #2, we suggest that you make a more nuanced conclusion explicitly stating that complementation is the likely explanation. It is important that you maintain a focus on describing the data.

We thank the editorial team for this decision and are happy to address the remaining concerns of Reviewer #2. Please see the detailed answers below and the corresponding changes in the track-changed version of the manuscript (lines 119-122).

**Response to Reviewers**

Reviewer #1: No further concerns. Interesting manuscript bringing important new information to the field.

Thank you very much!

Reviewer #2: As indicated in my previous review, this paper presents an interesting experimental setup that could help in the development of astrovirus culture and attenuated vaccines, but in my opinion does not report major advances in our understanding of astrovirus biology. For example, encephalitis-causing astroviruses have previously been cultured in neurons. <https://www.ncbi.nlm.nih.gov/pmc/articles/PMC6747723/>. The fact that serial transfer in a permissive cell line such as Huh7.5 leads to the accumulation of conditionally deleterious mutations is not surprising. Some of these mutations (but potentially many others) could be exploited for the development of live attenuated vaccines.

We are aware that other encephalitis-causing astroviruses have been cultured in neurons and have added this reference to the manuscript (#36) along with others. We do not claim that this study is the first demonstration of astrovirus infection in neurons, but previously responded that “To the best of our knowledge, this is the first report demonstrating the ability of MLB astroviruses to infect mature neurons and the first report demonstrating regions in the genome responsible for attenuation.” Indeed, there are many other regions to be discovered, and we look forward to these new findings.

Regarding my previous point 3, even if the MOI rises above 1 in secondary infection cycles, complementation is wiped out in each transfer. The rationale and interpretation of this passaging strategy remains therefore unclear.

We can see how the term “complementation” could cause confusion in the context of low MOI infections, as few cells will be infected with both virus genomes in the first instance. Many viruses passaged at high multiplicities of infection commonly accumulate defective-interfering (DI) particles – a well-known draw-back that we aimed to avoid in this study ([https://pubmed.ncbi.nlm.nih.gov/4356530](https://eur03.safelinks.protection.outlook.com/?url=https%3A%2F%2Fpubmed.ncbi.nlm.nih.gov%2F4356530&data=05%7C01%7Cvl284%40cam.ac.uk%7C757ab424ff604c7cb72908db5bc14494%7C49a50445bdfa4b79ade3547b4f3986e9%7C1%7C0%7C638204657852452137%7CUnknown%7CTWFpbGZsb3d8eyJWIjoiMC4wLjAwMDAiLCJQIjoiV2luMzIiLCJBTiI6Ik1haWwiLCJXVCI6Mn0%3D%7C3000%7C%7C%7C&sdata=DoM4m2GjodQboc0yoWDl4NeVzFjknu5zvbu%2FfNbREq8%3D&reserved=0); [https://pubmed.ncbi.nlm.nih.gov/25728309](https://eur03.safelinks.protection.outlook.com/?url=https%3A%2F%2Fpubmed.ncbi.nlm.nih.gov%2F25728309&data=05%7C01%7Cvl284%40cam.ac.uk%7C757ab424ff604c7cb72908db5bc14494%7C49a50445bdfa4b79ade3547b4f3986e9%7C1%7C0%7C638204657852452137%7CUnknown%7CTWFpbGZsb3d8eyJWIjoiMC4wLjAwMDAiLCJQIjoiV2luMzIiLCJBTiI6Ik1haWwiLCJXVCI6Mn0%3D%7C3000%7C%7C%7C&sdata=qHlR8vc3W6vSt9T9zMkj8w%2FoN1WzHdcxpNDjVy005hc%3D&reserved=0); [https://pubmed.ncbi.nlm.nih.gov/30245734](https://eur03.safelinks.protection.outlook.com/?url=https%3A%2F%2Fpubmed.ncbi.nlm.nih.gov%2F30245734&data=05%7C01%7Cvl284%40cam.ac.uk%7C757ab424ff604c7cb72908db5bc14494%7C49a50445bdfa4b79ade3547b4f3986e9%7C1%7C0%7C638204657852452137%7CUnknown%7CTWFpbGZsb3d8eyJWIjoiMC4wLjAwMDAiLCJQIjoiV2luMzIiLCJBTiI6Ik1haWwiLCJXVCI6Mn0%3D%7C3000%7C%7C%7C&sdata=2zrkg3RR%2BxGlEDtlFWS%2BHEyD%2FkMu%2FvR%2FCvfUI%2FIMPFE%3D&reserved=0)). We have clarified the text and added this explanation of why we chose a low MOI infection to the manuscript.

Reviewer #3: All comments have been adequately addressed, thank you and well done!

Thank you very much!

**Suggested editorial changes**

1. DATA POLICY:

You may be aware of the PLOS Data Policy, which requires that all data be made available without restriction: <http://journals.plos.org/plosbiology/s/data-availability>. For more information, please also see this editorial: <http://dx.doi.org/10.1371/journal.pbio.1001797>

Note that we do not require all raw data. Rather, we ask that all individual quantitative observations that underlie the data summarized in the figures and results of your paper be made available in one of the following forms:

1) Supplementary files (e.g., excel). Please ensure that all data files are uploaded as 'Supporting Information' and are invariably referred to (in the manuscript, figure legends, and the Description field when uploading your files) using the following format verbatim: S1 Data, S2 Data, etc. Multiple panels of a single or even several figures can be included as multiple sheets in one excel file that is saved using exactly the following convention: S1_Data.xlsx (using an underscore).

Done. Please see the track-changed file.

2) Deposition in a publicly available repository. Please also provide the accession code or a reviewer link so that we may view your data before publication.

Done. Both GenBank submissions are going live on 30^th^ of May – please see attached email confirmation (GenBank submissions.pdf).

Regardless of the method selected, please ensure that you provide the individual numerical values that underlie the summary data displayed in the following figure panels as they are essential for readers to assess your analysis and to reproduce it: 2EH, 3DEHI, 4D, 5DEFG, 6DEG, S1ACDEGH (We are aware that you provided the underlying data).

NOTE: the numerical data provided should include all replicates AND the way in which the plotted mean and errors were derived (it should not present only the mean/average values).

Done. The supplementary data file is updated accordingly.

**Please also ensure that figure legends in your manuscript include information on where the underlying data can be found, and ensure your supplemental data file/s has a legend.**

Done. The figure legends have been updated accordingly.

Please ensure that your Data Statement in the submission system accurately describes where your data can be found.

Done. The supplementary data file is updated accordingly.

2. BLOT AND GEL REPORTING REQUIREMENTS:

We require the original, uncropped and minimally adjusted images supporting all blot and gel results reported in an article's figures or Supporting Information files. We will require these files before a manuscript can be accepted so please prepare and upload them now. We need this for figure 2B.

Please carefully read our guidelines for how to prepare and upload this data: <https://journals.plos.org/plosbiology/s/figures#loc-blot-and-gel-reporting-requirements>

Done. Figure 2B was added to the updated S1_Raw_Images PDF file.

3. We suggest a more appropriate title for a Methods and Resources manuscript: "Identification of attenuation hotspots in human neurotropic astroviruses uncovers determinants of neurotropism and provides a platform for attenuated vaccine development". Please feel free to modify as you think fits better.

If possible, we would prefer to keep the original title. The development of the platform (reverse genetics and replicon system) described in this manuscript is not linked to attenuation hotspots, but was developed to characterise them instead. Adding this to the title in the correct phrasing makes it bulky and convoluted. We have discussed this with the co-authors and believe that the original title appears much better.

As you address these items, please take this last chance to review your reference list to ensure that it is complete and correct. If you have cited papers that have been retracted, please include the rationale for doing so in the manuscript text, or remove these references and replace them with relevant current references. Any changes to the reference list should be mentioned in the cover letter that accompanies your revised manuscript. We expect to receive your revised manuscript within two weeks.

We have added two additional references: one mentioned by Reviewer #2 (ref. #36), and another to further justify the choice of MOI (ref. #29). This is included in the cover letter.
